# Supplementary material for: Splice-Junction-Based Mapping of Alternative Isoforms in the Human Proteome
Source: Cell Rep. Author manuscript; Available in PMC 2020 Jan 15. (PMC6961840; doi:10.1016/j.celrep.2019.11.026)
Supplement: 3 [file NIHMS1546469-supplement-3.zip › DF2/PXD0010154/Esophagus-75-Q13683-LIPVPANSYFGFSIDSGK.pdf]

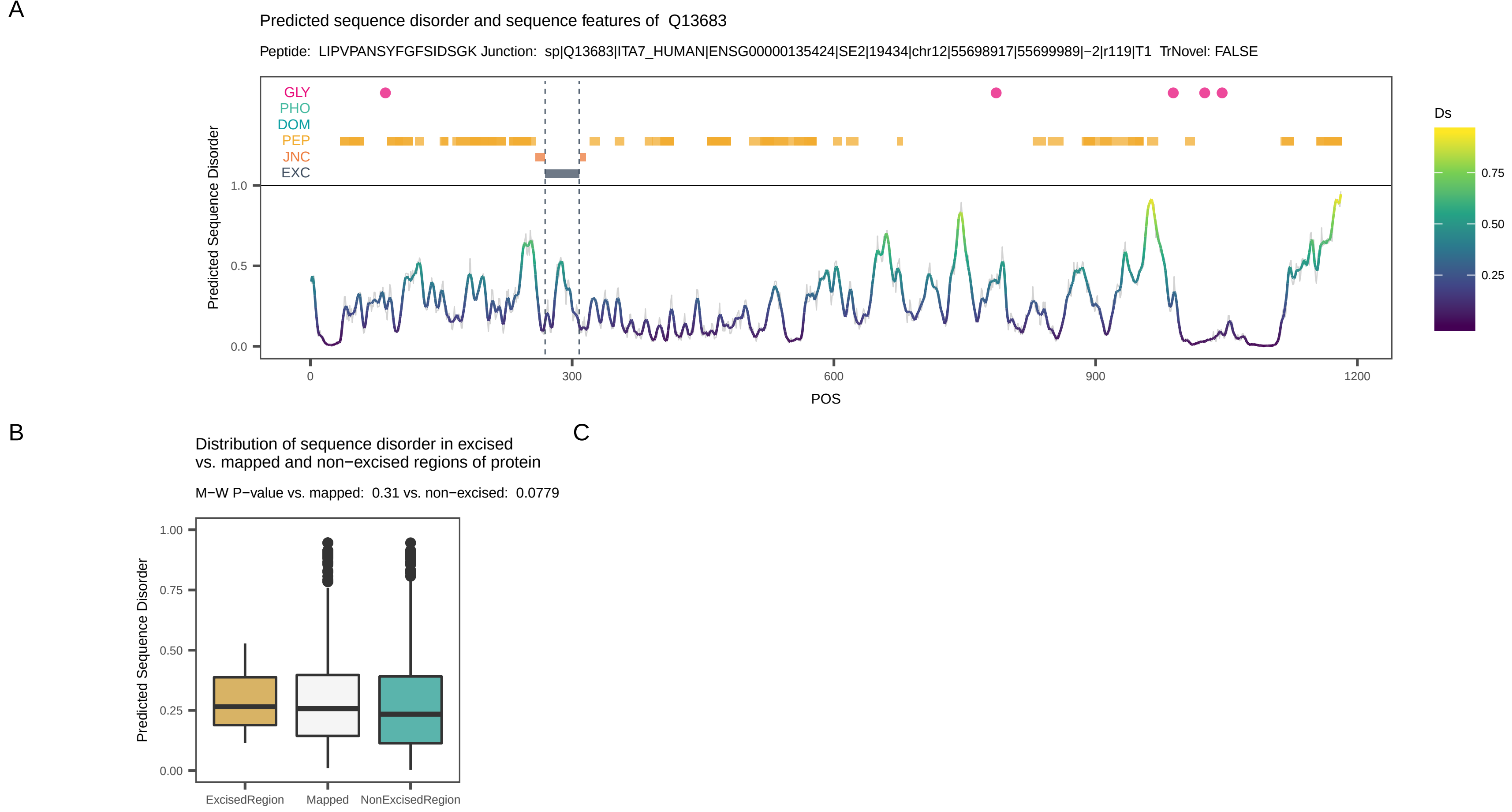
A
Predicted sequence disorder and sequence features of Q13683
Peptide: LIPVPANSYFGFSIDSGK Junction: sp|Q13683|ITA7_HUMAN|ENSG00000135424|SE2|19434|chr12|55698917|55699989|−2|r119|T1 TrNovel: FALSE
GLY
PHO
DOM
PEP
JNC
EXC
Predicted Sequence Disorder
1.0
0.5
0.0
0
300
600
900
1200
POS
Ds
0.75
0.50
0.25
B
Distribution of sequence disorder in excised
vs. mapped and non−excised regions of protein
M−W P−value vs. mapped: 0.31 vs. non−excised: 0.0779
Predicted Sequence Disorder
1.00
0.75
0.50
0.25
0.00
ExcisedRegion
Mapped
NonExcisedRegion
C
